# A post-gene silencing bioinformatics protocol for plant-defence gene validation and underlying process identification: case study of the *Arabidopsis thaliana NPR1*

**DOI:** 10.1186/s12870-017-1151-y

**Published:** 2017-11-23

**Authors:** Rosita E. Yocgo, Ephifania Geza, Emile R. Chimusa, Gaston K. Mazandu

**Affiliations:** 1 African Institute for Mathematical Sciences (AIMS), AIMS South Africa and AIMS Ghana, Cape Town, South Africa; 2 Biomathematics Division, Department of Mathematical Sciences, Stellenbosch University, Stellenbosch, South Africa; 3 Computational Biology Division, Department of Integrative Biomedical Sciences, Institute of Infectious Disease and Molecular Medicine, University of Cape Town, Medical School, Anzio Road, Observatory, Cape Town, 7925 South Africa; 4 Division of Human Genetics, Department of Pathology, Institute of Infectious Disease and Molecular Medicine, University of Cape Town, Medical School, Anzio Road, Observatory, Cape Town, 7925 South Africa

**Keywords:** Gene silencing, Plant defence gene discovery, Semantic similarity, *Arabidopsis thaliana*, *Pseudomonas syringae*

## Abstract

**Background:**

Advances in forward and reverse genetic techniques have enabled the discovery and identification of several plant defence genes based on quantifiable disease phenotypes in mutant populations. Existing models for testing the effect of gene inactivation or genes causing these phenotypes do not take into account eventual uncertainty of these datasets and potential noise inherent in the biological experiment used, which may mask downstream analysis and limit the use of these datasets. Moreover, elucidating biological mechanisms driving the induced disease resistance and influencing these observable disease phenotypes has never been systematically tackled, eliciting the need for an efficient model to characterize completely the gene target under consideration.

**Results:**

We developed a post-gene silencing bioinformatics (post-GSB) protocol which accounts for potential biases related to the disease phenotype datasets in assessing the contribution of the gene target to the plant defence response. The post-GSB protocol uses Gene Ontology semantic similarity and pathway dataset to generate enriched process regulatory network based on the functional degeneracy of the plant proteome to help understand the induced plant defence response. We applied this protocol to investigate the effect of the *NPR1* gene silencing to changes in *Arabidopsis thaliana* plants following *Pseudomonas syringae pathovar tomato* strain DC3000 infection. Results indicated that the presence of a functionally active *NPR1* reduced the plant’s susceptibility to the infection, with about 99% of variability in *Pseudomonas* spore growth between *npr1* mutant and wild-type samples. Moreover, the post-GSB protocol has revealed the coordinate action of target-associated genes and pathways through an enriched process regulatory network, summarizing the potential target-based induced disease resistance mechanism.

**Conclusions:**

This protocol can improve the characterization of the gene target and, potentially, elucidate induced defence response by more effectively utilizing available phenotype information and plant proteome functional knowledge.

**Electronic supplementary material:**

The online version of this article (doi:10.1186/s12870-017-1151-y) contains supplementary material, which is available to authorized users.

## Background

Plants are sessile organisms often subjected to several attacks and invasions from herbivorous and pathogenic organisms. Pathogenic organisms have been repeatedly reported to cause outbreaks on bean, cucumber, stone fruit, kiwi and olive tree, as well as on other crop and non-crop plants [1]. Plants respond to these attacks by switching on an array of defence pathways whose end products serve to limit the progression of invading pathogens. The innate response is the first stratum and earliest form of response reported. It involves interaction between pathogen associated molecular patterns (PAMP/MAMP) from the invading pathogen, and the plant’s membrane-localized pattern recognition receptors [2–4]. Downstream of this is the *hypersensitive response* (HR) which is highly specific and requires the recognition of specific avirulent genes from the pathogen by specific resistance (R) genes in the plant [5, 6]. Further downstream is a broad spectrum-type of response called *systemic acquired resistance* (SAR), a long-lasting defence response which primes the plant against future pathogenic attack [2, 7]. In 1999, Pieterse and Van Loon [8] highlighted the existence of yet another form of systemic response called *induced systemic response* (ISR), which can occur independently of the HR and SAR.

These strata of defence response pathways are generally governed by perturbations in the cells redox state, fluctuations in intracellular ionic concentration, activation/repression of kinases, synthesis of diverse signalling molecules, including *salicylic acid* (SA), *jasmonic acid* (JA), *ethylene* (Et), activation/repression of transcriptional co-regulators, such as the *non-expressor of pathogenesis-related 1* (*NPR1*), activation/repression of transcription factors, such as *TGA* and *WRKY*, and the ultimate expression/suppression of defence end products, such as pathogenesis-related (PR) proteins, which influences the growth and progression of the invading pathogens [9–14]. Understanding the biology of the plant protection and defence response may help in controlling the performance and survival of plants, which are very often faced with several types of stresses. The induced resistance response requires a coordinate action of many defence-related genes interacting within processes and signalling pathways to control down-stream responses during stress conditions. Computational modelling is therefore playing an additional role [15] in improving our understanding of plant defence and disease resistance mechanisms in a manner that is less costly, reduces redundancies and increases robustness. Such models offer attractive alternatives for the identification of gaps and opportunities in already characterized biological systems, and could eventually inform the design of more targeted molecular biology experiments [16].

*Pseudomonas syringae* (*P. syringae*) is a rod shaped Gram-negative bacterium, a major bacterial leaf pathogen that causes diseases in a wide range of plant species with visible symptoms [17, 18]. A specific variety of *P. syringae pathovar tomato* strain DC3000 (*Pst*-DC3000), is known to infect DC3000 *tomato* and *Arabidopsis thaliana* plants [19], using the plant leaf surface to negotiate its entry into the plant. Both *Arabidopsis* [20] and *Pst*-DC3000 [19] have been sequenced and are genetically tractable, economically and environmentally convenient, and constitute a model system in research for studying molecular pathogenesis and dynamics of plant-pathogens interactions [21]. Interestingly, *Arabidopsis thaliana* shares high similarity with high agronomic value crops, such as rice with similarity of 0.71 [22], suggesting that the findings related to *Arabidopsis thaliana* can be assessed to determine whether they could be applied to these different plants.

**Fig. 1:**
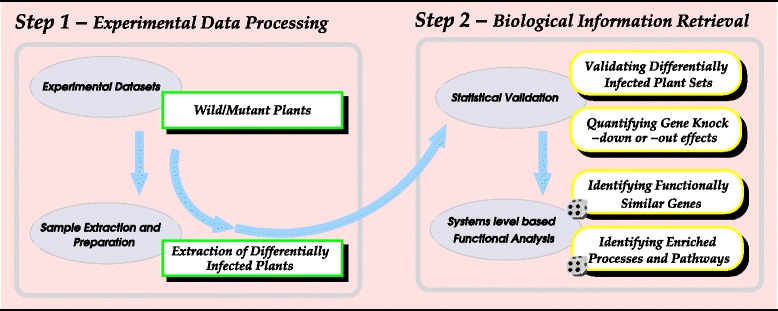
The post-gene silencing bioinformatics (post-GSB) scheme. This describes the two steps of the model as described in the scheme. The first step consists of producing experimental data sets and using the closeness scores between plants to extract the set of differentially infected plants to be considered for further analyses. The second step initially focuses on statistical analyses checking whether there is any difference between the two data sets: wild type and *npr1* gene mutant plants. If this difference is significant, then the Gene Ontology process annotation based functional analysis is performed to identify proteins, enriched processes and pathways contributing to the *NPR1*-based plant defence mechanisms

In this study, we used *NPR1* (or *SAI1* for salicylic acid-insensitivity or *NIM1* for non-inducible immunity) as a reference due to the available experimental data and its central role in SAR signalling pathway, a broad-spectrum resistance protecting against several pathogenic attacks [23], which activates antimicrobial genes products in both the model plant *Arabidopsis thaliana* and a diverse number of food crops [4, 24–32], including canola, cabbage, broccoli, tobacco, tomato, potato, corn. *NPR1* is also involved in the regulation of signalling pathways like abscisic acid (ABA) [33], which, as JA- and Et-dependent activation of defense responses resulting from ISR [17], offers resistance to necrotrophic pathogens. We propose a post-gene silencing bioinformatics (post-GSB) protocol, a two-step approach (see Fig. 1), which can be used to efficiently analyze plant differential responses based on leaf spore count or expression level experimental data sets. This post-GSB protocol introduces the closeness score concept between plants to extract differentially infected plants in a population of *Arabidopsis thaliana* wild type and *npr1* mutant plants infected with *Pst*-DC3000 used in further analyses. It uses a Gene Ontology (GO) based semantic similarity model to identify proteins collaborating with *NPR1* as well as enriched biological processes involved in the defence response and elucidate these process occurrence sequences. This new model manages eventual uncertainty of data, potential noise inherent in the experiment and redundancy from the semantic-based GO structure to effectively assess contributions of and validate plant-defence genes, and predict plant-defence mechanisms.

## Methods

The study was divided into two parts, the first was to check whether *NPR1* plays an important role in plant protection and secondly to perform functional analyses to predict the biological mechanisms of the *NPR1*-based defence response.

### Experimental data from *Arabidopsis* wild type and *npr1* mutant plants

Data analyzed in this study was derived from a study conducted at the John Innes Centre (England) in 2010 [32] and consisted of data from wild type (Wt) and *npr1* mutant *Arabidopsis thaliana* Columbia (Col – 0) plants 48 hours post infection with *Pst*-DC3000 [34]. These plants were grown and inoculated under controlled greenhouse conditions (23^°^ C, 10 h day/14 h dark regime and a relative humidity of 65±5*%*) in the John Innes Centre in England [32]. *Pst*-DC3000 bacteria strain was cultured as described in [34] using King B’s media [ 20 *gL* ^−1^ proteose peptone (w/v), 1.5 *gL* ^−1^ di-potassium hydrogen phosphate (w/v), 1.5 *gL* ^−1^ magnesium sulphate (w/v), 1.5*%* glycerol (v/v) and 1.2*%* bacterio agar (w/v)] supplemented with 50 *mgL* ^−1^ kanamycin. The bacteria cells were re-suspended in a 10 *mM* *MgCl*_2_ solution and the concentration adjusted to 5×105 colony forming unit *cfu*.*mL* ^−1^. For each plant to be inoculated, three leaves were hand-infiltrated using a 1 *mL* needless syringe containing either a 10 *mM* *MgCl*_2_ solution or an inoculum of *Pst*-DC3000 (5×105 *cfu*.*mL* ^−1^). Bacteria growth was measured as spore counts 48 h post treatment using 8 *mm* leaf discs excised from the treated plants. Measurements were conducted using a FB12 luminometer with a single photon counter [32, 34]. For adequate representation and statistics purposes, the experiment was conducted three times using a total of 60 plants (three infected leaves per plant) per genotype (see raw data in Additional file 1).

### Sample extraction and analysis

**Fig. 2:**
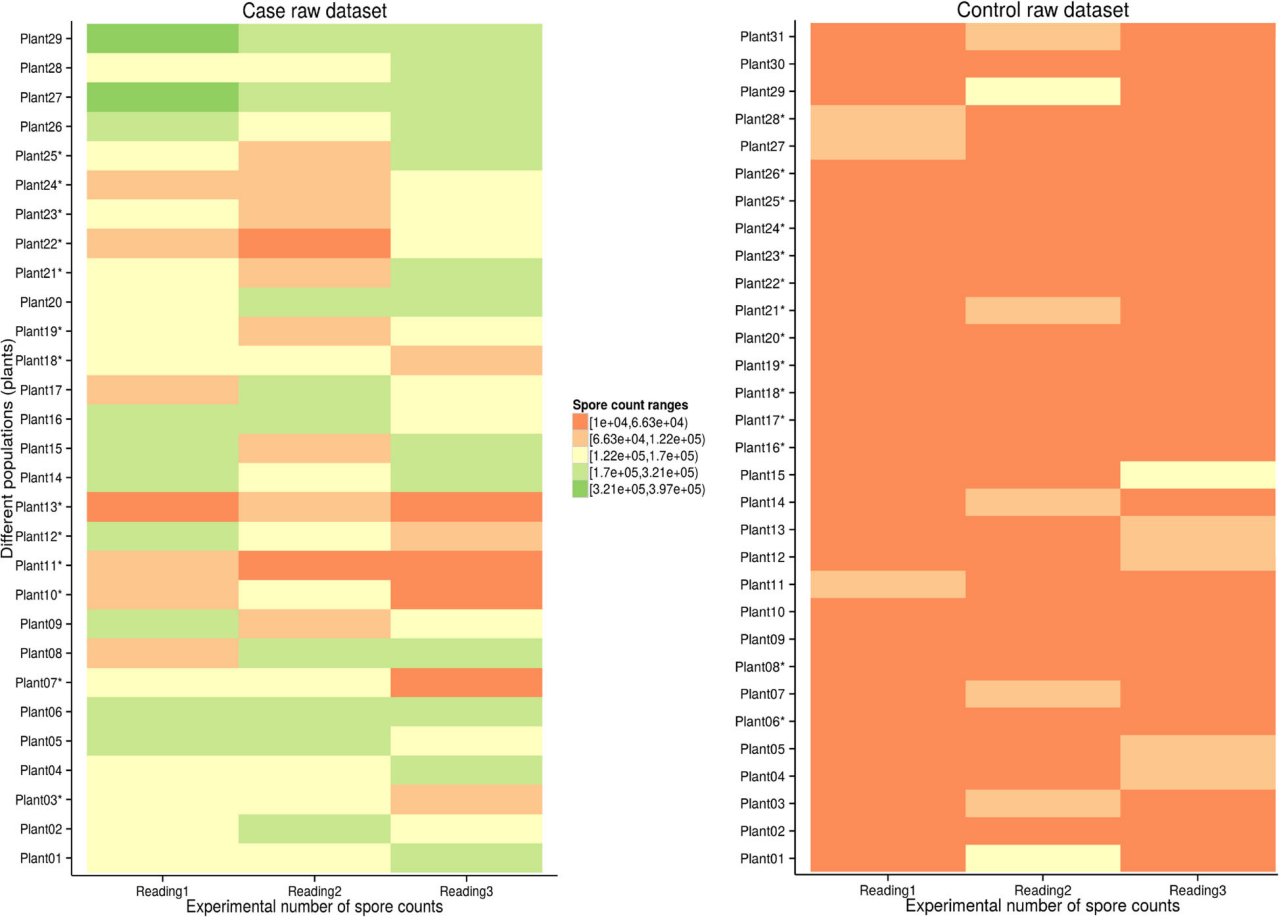
Plotted heat-maps of spore counts for the case and control raw datasets. These plots (cases and control datasets on left and right sides, respectively) show variations of leaf spore counts from relatively lower (dark orange) to higher (dark green) counts as an indication of low and high levels of infection. An asterix on a given plant identifier indicates that the plant is among differentially infected plants within a dataset

Data from a total of 31 *Arabidopsis* Wt plants (controls) and 29 *npr1* mutant plants (cases) inoculated with *Pst*-DC3000 were used for further analysis. Figure 2 is a graphical representation of the spore count data to visually assess the distribution of these spore counts. Noticing that there was a distribution pattern, samples were further analysed in three main steps: (1) computing the closeness score of each individual in a sample and ranking these individuals based on these scores; (2) extracting the list of differentially infected plants using Pearson *χ* ^2^ scores based on aggregated closeness scores; and (3) statistically measuring the significant difference among the two phenotypes.

#### Computing individual closeness scores

Generally, a set of plants within each group consists of a mix of phenotype tendency associated with different levels (moderate or high) of infection observed through plant spore counts. Since at a higher level of infection, it may not be evident to distinguish between the two groups under consideration, there is a need for a scoring scheme which can appropriately classify plants with moderate level of infection, referred to as differentially infected plants, based on plant spore counts. Here we set up a closeness score approach to measure the length of the line-segment joining the plant phenotypes using spore count vectors, enabling the selection of differentially infected plants. The individual closeness score of an individual (plant) *ı*, denoted ICS(*ı*), in a sample is computed as follows:

1 $$ \text{ICS}(\imath)= \sum\limits_{\jmath=1}^{n} \mathcal{S}_{\imath\jmath}  $$

where *n* is the size of the sample (number of individuals in the sample) and $\mathcal {S}_{\imath \jmath }$ is the similarity score between individuals *ı* and *ȷ* calculated using the Manhattan metric defined on a vector of spore counts for three different readings and converted to similarity measure following the Resnik-edge-based approach [35, 36]. This similarity score $\mathcal {S}_{\imath \jmath }$ is given by:

2 $$ \mathcal{S}_{\imath\jmath} = 2\times \delta\left(x_{\imath}\right) - d\left(x_{\imath}, x_{\jmath}\right)  $$

with *x*_*k*_ a spore count vector of individual *k*, *δ*(*x*_*ı*_)= max{*d*(*x*_*ı*_,*x*_*ȷ*_):*ȷ*=1,…,*n*} and *d*(*x*_*ı*_,*x*_*ȷ*_) the distance between spore count vectors of the two individuals based on the Manhattan metric defined on a vector of spore counts, computed as follows:

3 $$ d\left(x_{\imath}, x_{\jmath}\right) = \min\left\{\sum_{k=1}^{n}\left|x_{\imath k}-x_{\jmath p(k)}\right|: p\in \mathcal{P}\right\}  $$

where $\mathcal {P}$ is the set of all permutations of the ordered set *S*={1,2,…,*n*} of *n* readings in the sample. The individual or plant with the highest ICS is referred to as reference individual or plant, whose similarity scores to other plants are used to extract the set or list of plants of interest for further statistical analyses.

#### Extracting sets of differentially infected plants in different samples

In order to minimize the impact of potential biases which may emanate from highly infected plants, we design a model for selecting sets of differentially infected plants in which spore counts based phenotypes are significantly associated with moderate level of infection in different samples based on closeness or similarity score described in the subsection above. Using similarity scores between reference plant, denoted *d*, and all plants in a sample, the list of these plants were ranked from the highest to the lowest scores. The ranked plant list is then used to compute a Pearson *χ* ^2^ score for each plant subset, reflecting the tendency of plants in a particular set to occur towards the extremes of the list. The Pearson *χ* ^2^ score of a subset containing plants re-indexed from *ℓ* to *m*, denoted $\chi _{P}^{2}\left (\ell, m\right)$, is given by:

4 $$ \chi_{P}^{2}\left(\ell, m\right) = \sum\limits_{\jmath=\ell}^{m} \frac{\left(\mathcal{S}_{d\jmath}-\mu_{\ell m}\right)^{2}}{\mu_{\ell m}}  $$

which is known to approximate a *χ* ^2^-distribution with (*m*−*ℓ*) degree of freedom (dof) and where their expected value *μ*_*ℓm*_ represents aggregated score (ES) of the extracted subset and estimated as follows:

5 $$ \mu_{\ell m} = \text{ES}\left(m-\ell +1\right)= \sum\limits_{\jmath=\ell}^{m} \mathcal{S}_{d\jmath}  $$

We identified *s* top individuals as differentially infected with *s* the index fold-change $\chi _{P}^{2}$. Assuming that the ranked list contains *n* individuals, *s* (*s*≤*n*) is the smallest index satisfying the following inequality:

6 $$ \frac{\chi_{P}^{2}\left(1, s\right)-r*\chi_{P}^{2}\left(s+1, n\right)}{\sqrt{1+r^{2}}} > 0  $$

where

7 $$ r = \sqrt{\frac{s-1}{n-s-1}}  $$

which is the ratio of standard deviations of $\chi _{P}^{2}\left (1, s\right)$ and $\chi _{P}^{2}\left (s+1, n\right)$, and set to 1 if *s*=1.

The significance of the aggregated score, ES(*s*), of the subset of differentially infected plants was assessed using sample randomization. So, we randomly selected 1000 independent subsets (same size with the set of differentially infected) of plants and compute ES of each subset and then perform the Shapiro-Wilk test under the null hypothesis that the generated sample is drawn from a normal distribution. Following the rejection or the acceptance of the null hypothesis, we perform the Wilcoxon- or the T-test to check whether the identified set of differentially infected individuals is more than expected by chance.

#### Statistical analyis of *npr1* mutant and wild-type plant based disease symptoms

The contribution of *NPR1* protein to the plant protection during infection as the defence co-regulator is statistically assessed on sets of differentially infected plants extracted from the two population samples (mutant/case and wild/control). We test for the differences between the mutant and the wild type plants using parametric or non parametric statistics given that agreements with respects to the violations of the assumptions are met. Thus, the normality and variance homogeneity assumptions of the two samples are tested using the Shapiro-Wilk and Bartlett tests, respectively. In order to satisfy these assumptions for datasets which are characterized by extreme non-normality or heterogeneity of variance, we apply a data transformation [37, 38] based on the Fisher-Pearson coefficient of skewness [39] to bring the data into greater agreement with the normality and variance homogeneity assumptions. Depending on whether normality and variance homogeneity assumptions are met or not, we performed the analysis of variance (ANOVA) method or the non-parametric Kruskal-Wallis rank sum test.

### Bioinformatics analysis of potential *NPR1*-based plant defence mechanisms

We used the Gene Ontology (GO) [40, 41] and the protein GO Annotation (GOA) mapping provided by the UniProtKB-GOA project [42–44] to reveal the potential *NPR1*-based regulatory network using enriched biological processes and pathways in which a set of protein targets are involved. The complete set of GO data and protein-GO term associations were extracted from the GO and GOA databases, respectively, accessed on the 11th of October, 2016. For the pathway enrichment analysis, we use the Kyoto Encyclopeadia of Genes and Genomes (KEGG) datasets. The whole *Arabidopsis thaliana* pathway dataset was extracted from the KEGG database at http://www.genome.jp/kegg/.

#### Identification of other putative proteins participating to the plant defence response

Proteins perform an astonishing range of biological functions in an organism by collaborating in pathways and processes, and interacting with the cellular environment in some way to promote the cell’s growth and function [45, 46]. This argues that the induced resistance response requires concerted biological action of many genes involved in diverse processes or defence signalling pathways. There are various computational approaches for identifying proteins co-working or involved in a similar process, which, in general, rely on a *priori* knowledge of protein functional features. This functional knowledge can be either a protein-protein interaction (PPI) network, in which case, these proteins interact and influence each other in the same sub-network or protein functional annotations, in which case, these proteins are involved in semantically similar processes or in the same pathway. In this study, since we are looking at the *NPR1* biological role and functional annotations, we use protein biological process dataset to identify putative proteins which are functionally similar to *Arabidopsis NPR1* by quantifying functional similarity between proteins based on sets of strict non-redundant processes annotating proteins under consideration [47].

Given two proteins *p* and *q*, the functional similarity between *p* and *q*, BMA(*p,q*), is computed using the Best Match Average model [48–51] as follows:

8 $$ \text{BMA}\left(p,q\right) = \frac12\left(\frac1n{\sum\limits_{s\in T_{p}}\mathcal{S}\left(s, T_{q}\right)}+\frac1m{\sum\limits_{s\in T_{q}}\mathcal{S}\left(s, T_{p}\right)}\right)  $$

where *T*_*r*_ is a set of process terms annotating a given protein *r* and *n*=|*T*_*p*_| and *m*=|*T*_*q*_| are the number of processes terms in these sets. $\mathcal {S}\left (s, T_{r}\right) = \max \left \{\mathcal {S}\left (s,t\right): t \in T_{r}\right \}$ with $\mathcal {S}\left (s,t\right),$ the semantic similarity score between process terms *s* and *t* computed using the GO-universal metric [48]. It is worth noting that semantic similarity measure has proved its effectiveness to solving biological issues related to the Gene Ontology annotation-based knowledge discovery, including predicting and validating protein-protein interaction (PPI) [52]. With curated PPIs, which are still relatively scarce, high-throughput biology experiments and computational methods producing high rate of false positive PPIs [53], and the lack of appropriate techniques to address these shortcomings [45], semantic similarity-based approach is more effective, providing a classification indicator of PPIs in the absence of reliable information.

#### Identifying enriched *Arabidopsis* defence response processes and pathways

For process or pathway enrichment analysis, we used hyper-geometric test adjusted using the Bonferroni multiple testing correction. For each process or pathway, the *p*-value was calculated using its frequencies of occurrence in the reference dataset (*Arabidopsis thaliana* proteome) and target set, which composed of proteins identified to be functionally similar to *NPR1*. Thus, this *p*-value, which is the probability of observing at least *s* proteins from a target gene set of size *n* by chance, knowing that the reference dataset contains *m* annotated genes out of N genes, is given by the following formula [54, 55]:

9 $$ P\left[X\geq s\right] = 1 - \sum\limits_{k = 0}^{s-1} \frac{\binom{m}{k}\binom{N-m}{n-k}}{\binom{N}{n}}  $$

where the random variable *X* is the number of genes involved in the GO process or participating to the pathway under consideration within a given target gene set contributing to plant defence response. To account for relationships between GO terms in the GO structure, we used the concept of the GO term semantic similarity score [55] and the frequency of occurrence *f*(*t*) of the target-associated process *t* in a set of proteins *P* is given by:

10 $$ f(t) = \sum\limits_{q \in P}\delta_{q}(t)  $$

where *δ*_*q*_ is the *q*-function indicator given by

11 $$ \delta_{q}(t) =\left\{ \begin{array}{ll} 1 & \text{if}~ \mathcal{S}_{q}(t) > \varepsilon\\ 0 & \text{otherwise} \end{array} \right.  $$

where *ε*≥0 is the agreement level or customized agreement at which a GO process is considered to be a possible annotation of the protein *q* and $\mathcal {S}_{q}(t)=\mathcal {S}\left (t, T_{q}\right)$, representing the semantic similarity degree at which a related term is considered to semantically reflect in the specification of the term *t* [47]. Here, *ε* was set to 0 for *t*∈*T*_*q*_ or $t\in \mathcal {A}_{q}$, and to 0.7 for $t\in \mathcal {C}_{q}$ where $\mathcal {A}_{q}$ and $\mathcal {C}_{q}$ are sets of ancestors and descendants of processes in *T*_*q*_, respectively. The *p*-value of each process term was adjusted using the Bonferroni multiple testing correction.

## Results and discussion

In the first part of the post-GSB framework developed in this study, we have designed a spore count closeness scoring approach. This enables the identification of differentially infected plants used to ascertain the role of *NPR1* in *Arabidopsis* plant defence response with bacteria spore count data collected from *Arabidopsis* Wt and *npr1* mutant leaves 48 h post *Pst*-DC3000 infection as described in the “Methods” section. We then predicted the potential *NPR1*-based regulatory network based on the identified set of putative proteins, enriched biological processes and pathways which participate in plant defence response.

### *NPR1* protein is required for *Arabidopsis* defence response

The heat map representation of the raw experimental spore count data from both data sets in Fig. 2 revealed that spore distribution patterns in the *Arabidopsis* Wt infected leaves are more closely similar (see Fig. 2 b) in comparison to those of the mutant (Fig. 2 a). This is in agreement with previous results which showed a significant difference in spore numbers between the two phenotypes (data not shown) [32]. In a study of Wt and *npr1* mutant plants by Cao et al. [56], it was observed that infection spread event to uninfected parts of the leaf is quite rapid due to its high level of susceptibility. To eliminate the possibility that the presence of highly infected plants in the mutant population is contributing significantly to the observed differential response found in this study, we statistically tested the difference between the two groups of plants on specific sets of plants from these two groups. These sets contains only differentially or moderately infected plants in order to keep out the highly infected plants within the two phenotype groups using the closeness scoring approach. Outputs generated 13 moderately infected *npr1* mutant plants, and 14 moderately infected *Arabidopsis* Wt plants which were used for further downstream analysis (Fig. 2, plants marked with an asterisk). Note that these identified sets of differentially infected plants for both *npr1* mutant and wild-type plants are statistically significant with *p*-values < 2*e*−16.

The graphical representation (Q-Q plots) of the above Wt plant sub-data indicated that this sub-data was not normally distributed (see Additional file 2: Figure S1) with the Shapiro-Wilk test *p*–value of 0.000152≤0.05, thereby leading to a two-step statistical analysis process. Firstly, we tested for statistical differences using the non-parametric Kruskal-Wallis rank sum test. Results from this test indicated that within phenotypes, there was no significance difference (*p*–value =0.70>0.05), but, between the two phenotypes, there was a significant difference in the number of bacteria spores (*p*–values =4.551*e*−12≤0.05). Noting that non-parametric tests have weaker statistical power in comparison to parametric tests, and to further eliminate the possibility of introducing a type II error [57], we applied a parametric approach to increase the robustness of our analysis.

In order to bring different data subsets into agreement with the normality and homogeneity of variance (equal population variance) assumptions, these data subsets were log transformed (see Additional file 2: Figure S2) and scaled using their standard deviations. Note that the choice of log10 transformation was guided by the Fisher-Pearson skewness coefficient [37, 38] of the Wt dataset with a value of 1.46524, suggesting that its distribution was highly skewed (substantially positive skewness) [39]. The Shapiro-Wilk normality test and the Bartlett variance homogeneity *p*–values were greater than 0.1169 and 0.98 for both Wt and *npr1* mutant data subsets. Results from our ANOVA using this log transformed data subsets still confirmed that there was no significance difference within data subsets (*p*–value =0.481), but there existed a significant difference in spore numbers between both phenotypes (*p*–value < 2*e*−16) with the measure of association *ω*=0.98893. This indicates that 99% of the variability between Wt and *npr1* mutant samples may be attributed to whether the *NPR1* protein is functionally active or not, providing evidence that *NPR1* plays a critical role in plant defence response.

In summary, we applied the Pearson *χ* ^2^-based plant closeness scoring scheme in order to extract specific sets of plants in populations under consideration to be used for further statistical inferences. These experimental data subsets showed evidence that a functional *NPR1* is required to limit bacteria growth in *Arabidopsis* plants. This finding correlates with results from previous studies that have equally demonstrated that the presence of a functional *Arabidopsis NPR1* is important in limiting pathogen growth in infected plants [56, 58, 59], playing a key role in the SAR signalling pathway [23, 58, 60], the broad-spectrum type of response providing defence against secondary pathogens after a primary attack. This Pearson *χ* ^2^-based plant closeness scoring approach enables the use of more relevant data subsets in further analyses, thus limiting the effect of the eventual uncertainty of these datasets and potential noise inherent in the biological experiment used. This provides an increased robustness of statistical analyses and stronger validation of inferences from experimental datasets.

### Elucidating potential *NPR1*-based enriched process regulatory network

Similar to other organisms, plants combat the adverse effects of stressors by activating or deactivating various processes and pathways. We therefore applied a semantic similarity based computational model to gene products that are functionally similar to *NPR1* and identify enriched plant defence biological processes using protein GO annotation mapping from the GOA-UniProtKB dataset [42, 44] with the reviewed proteins in the *Arabidopsis* proteome retrieved from the UniProt database [61]. Furthermore, we used the *Arabidopsis* KEGG pathway dataset to retrieve enriched pathways participating to the *NPR1*-based regulation of *Arabidopsis* defence response and elucidated the GO-based regulatory network triggered by *NPR1*.

#### Identification of *NPR1*-associated plant defence proteins

**Fig. 3:**
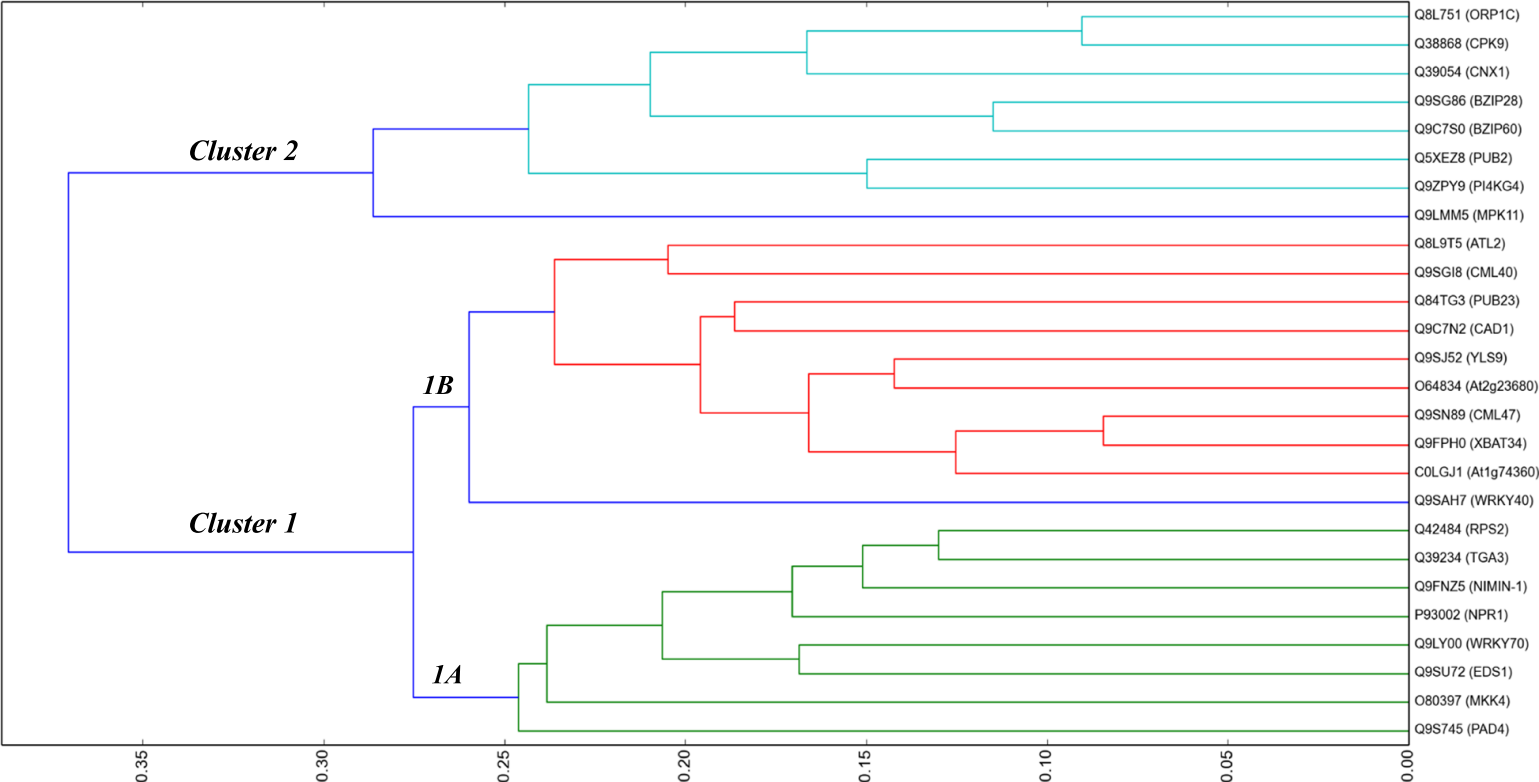
Functional similarity between *NPR1* and other associated proteins. A dendrogram showing the relatedness of 26 reviewed proteins in *Arabidopsis thaliana* proteome. This was obtained by applying hierarchical agglomerative clustering algorithm on the set of annotated proteins related to *NPR1* using a distance retrieved from functional similarity scores and represented on the horizontal axis

In general, defence mechanism is driven by several gene products that act dependently or independently. In order to identify *NPR1*-associated plant defence proteins, we computed the functional similarity scores between *NPR1* and other 20 545 annotated proteins [62] out of 51874 found in the complete list of proteins in the *Arabidopsis* proteome. In order to avoid over-estimating functional similarity scores between proteins [52], which is induced by the redundant processes from parent-child relations from the GO structure [47], we used the set of filtered non-redundant processes in which proteins are involved. Results indicated that 30 proteins shared high functional similarity to the *Arabidopsis NPR1* (Table 1) of which, 25 were reviewed or manually curated (in Table 1; protein with status *✓*). A hierarchical clustering representation of these 25 annotated proteins together with the *Arabidopsis* *NPR1* protein, which shows functional relatedness between these 26 reviewed proteins, produced two main clusters (Fig. 3) with Cluster 1 comprising of two sub clusters (1A and 1B). Sub-Cluster 1A contains proteins closest to *NPR1* (at the maximum distance of approximately 0.245) and distant at 0.275 from Sub-Cluster 1B, while proteins in Cluster 2 were the farthest subgroup being distant at 0.375 maximum from Cluster 1. Proteins in the sub-cluster 1A comprised of the *NPR1/NIM-1* interacting protein (*NIMIN-1*), transcription factor proteins (*TGA3, WRKY70*), Resistance to *P. syringae 2* (*RPS2*), two Enhanced Diseases Susceptibility proteins (*EDS1, PAD4*) and *Mitogen-activated protein kinase kinase 4* (*MKK4*). Those in the sub-cluster 1B comprised of *WRKY40, AT1G74360, XBAT34*, the putative *calmodulin-like protein 47* (*CML47*), *AT2G23680*, the *Yellow Leaf-Specific protein 9* (*YLS9*), *Constitutively Activated Cell Death 1* (*CAD1), PUB23, CML40*, and *ATL2*. Proteins in cluster 2 comprised of *Mitogen-activated Protein Kinase 11* (*MPK11*), *Phosphoinositide 4-Kinase Gamma 4* (*PI4KG4*), *PUB2*, two basic *Leu zipper* (*BZIP*) transcription factors (*BZIP60* and *bZIP28*), *Molybdopterin biosynthesis protein* (*CNX1*), *Calcium-dependent Protein Kinase 9* (*CPK9*), and *Oxysterol-binding Protein-Related Protein 1C* (*ORP1C2*).

**Table 1:** Proteins highly similar to *NPR1* or *Non-inducible immunity protein 1* (*NIM1*)

Entry name	Gene names	Status	Protein name
Q9LY00	*WRKY70* or *At3g56400*	*✓*	*Probable WRKY transcription factor 70 (WRKY DNA-binding protein 70)*
Q9LMM5	*MPK11* or *At1g01560*	*✓*	*Mitogen-activated protein kinase 11 (AtMPK11) (MAP kinase 11)*
O64834	*At2g23680* or *F26B6.33*	*✓*	*Cold-regulated 413 plasma membrane protein 3 (AtCOR413-PM3)*
Q9ZPY9	*PI4KG4* or *PI4KGamma4*	*✓*	*Phosphatidylinositol 4-kinase gamma 4 or At2g46500 (AtPI4Kgamma4) (PI4K gamma 4)*
Q39054	*CNX1* or *At5g20990* or *F22D1.6*	*✓*	*Molybdopterin biosynthesis protein CNX1*
Q5XEZ8	*PUB2* or *At5g67340*	*✓*	*U-box domain-containing protein 2*
Q9SJ52	*YLS9* or *NHL10* or *At2g35980*	*✓*	*Protein YLS9 (Protein NDR1/HIN1-LIKE 10)*
Q9SGI8	*CML40* or *At3g01830*	*✓*	*Probable calcium-binding protein CML40*
Q9FNZ5	*NIMIN-1* or *At1g02450*	*✓*	*Protein NIM1-Interacting 1 (NIMIN-1)*
Q38868	*CPK9* or *At3g20410*	*✓*	*Calcium-dependent protein kinase 9*
Q9C7SO	*BZIP60* or *At1g42990*	*✓*	*bZIP transcription factor 60 (AtbZIP60)*
O80397	*MKK4* or *At1g51660*	*✓*	*Mitogen-activated protein kinase kinase 4 (AtMKK4)*
Q9L9T5	*ATL2* or *At3g16720*	*✓*	*RING-H2 finger protein ATL2 (Protein Arabidopsis Toxicos En Levadura 2)*
Q9S745	*PAD4* or *EDS9* or *At3g52430*	*✓*	*Lipase-like PAD4 (Protein Enhanced Disease Susceptibility 9)*
Q42484	*RPS2* or *At4g26090*	*✓*	*Disease resistance protein RPS2 (Resistance to P-syringae protein 2)*
Q9SN89	*CML47* or *At3g47480*	*✓*	*Probable calcium-binding protein CML47*
Q9SU72	*EDS1* or *EDS1A* or *At3g48090*	*✓*	*Protein EDS1 (Enhanced Disease Susceptibility 1)*
Q8L751	*ORP1C* or *At4g08180*	*✓*	*Oxysterol-binding protein-related protein 1C*
Q39234	*TGA3* or *BZIP22* or *At1g22070*	*✓*	*Transcription factor TGA3 (AtbZIP22)*
Q9SAH7	*WRKY40* or *At1g80840*	*✓*	*Probable WRKY transcription factor 40 (WRKY DNA-binding protein 40)*
COLGJ1	*At1g74360* or *F1M20.4*	*✓*	*Threonine-protein kinase*
Q84TG3	*PUB23* or *At2g35930*	*✓*	*Plant U-box protein 23*
Q9FPHO	*XBAT34* or *At4g14365*	*✓*	*Putative E3 ubiquitin-protein ligase XBAT34*
Q9SG86	*BZIP28* or *At3g10800*	*✓*	*bZIP transcription factor 28 (AtbZIP28)*
Q9C7N2	*CAD1* or *At1g29690*	*✓*	*MACPF domain-containing protein CAD1 (Protein Constitutively Activated Cell Death 1)*
Q9C9H6	*RLP11* or *At1g71390*	✗	*Putative disease resistance protein (Receptor like protein 11)*
Q9SJQ8	*At2g36470*	✗	*Expressed protein (Uncharacterized protein)*
Q8RXN8	*At5g42050*	✗	*Development and Cell Death (DCD)*
Q8GYH5	*ANK* or *At5g54610*	✗	*Ankyrin repeat family protein*
Q9LN03	*At1g08050*	✗	*C3HC4-type RING finger-containing protein*

The gene names are given in the second column whilst the full protein name as downloaded from the UniProt database [61] is given in the fourth column. A gene can have more than one name, but throughout this study we use the first names of each gene. The third column indicates whether the protein has been reviewed (i.e., manually curated: marked *✓*) or not (✗)

An in-depth literature review demonstrated that these functionally related proteins could contribute in the same pathway as *NPR1* or independently to either positively or negatively drive defence response to pathogens and elicitors. For instance, calcium signalling is known to be associated to the innate response, HR and SAR, acting upstream of the pathogen-induced SA-signalling pathway to favor the activation of *NPR1* and associated downstream activities, such as *TGA* binding, to inhibit pathogen growth [63–65]. The calcium signalling triggers the activation of a set of Mitogen protein kinase (*MPK*) contributing to the plant innate immunity related cell death [66] and the MAMP defence [67], which is also an innate-form of defence response, and to other processes that stimulate ROS production during plant pathogen attack [68]. In this study, two *MPK* proteins, namely *MPK11* (Cluster 2) and *MKK4* (Cluster 1A) which is an important mediator of plant response to osmotic stress [69], were found to be closely related to *NPR1*. Proteins belonging to the EF hand super family have been shown to harbour motifs that are important for calcium sensing [70, 71]. Typical examples of proteins within this family are the *calmodulin (CaM)-like proteins* (*CML*) and *Calcium protein kinase* (*CPK*) [70, 72, 73] whose members: *CML47, CML40, CPK9* identified in this study belonged to Clusters 1A and 2. Moreover, two other proteins identified, *NDR1* (*YLS9*) in Cluster 1B and *EDS1* in Cluster 1A, are known to mediate the Effector Triggered Immunity through specific R genes [74, 75]. As illustration, *EDS1* was found to be a regulatory gene controlling down-stream defence gene expression in *Arabidopsis* [76], targeting R genes with *TIR-NB-LRR* motifs required for the recognition of *Pst*-DC3000 avrRps4 proteins [75, 77, 78]. R genes, such as *RPS2* (Cluster 1A), which recognizes *Pst*-DC3000 avrRpt2, are targeted by *NDR1* and harbour *LZ-NBS-LRR* motifs [74, 75, 77, 78]. *YLS9*, an *NDR1/HIN1*-like 10 (*NHL-10*) identified in our study has been implicated in HR and is inducible by several *CPKs* [79], thereby suggesting its action downstream of the calcium signalling pathway.

As pointed out previously, proteins functionally related to *NPR1* may contribute to the plant defence mechanism degradation. For example, *CAD1* (Cluster 1A) has been implicated in the repression of the *programmed cell death* (*PCD*) pathway mediated by SA and this negative function of *CAD1* is inhibited under stress conditions to favor the *PCD* pathway [80]. However, even under undisturbed conditions, plant proteins undergo proteolysis as a strategy to effectively turnover its protein reservoir and to ensure for efficient functioning of biological processes through an ubiquitination process [81, 82]. The *NPR1* protein, for instance, which can switch between two conformational states (oligomeric and monomeric) or two cell compartments (cytosol and nucleus), is a typical example of a protein whose nuclei-localized monomers undergo *proteolysis* through a *CUL-3*-based *E3 ligase ubiquitination* process to ensure for the effective expression of its target genes [59, 83, 84]. Our analysis identified five putative ubiquitin-like proteins - *XBA34, PUB23, PUB2, ATL2, PI4KG4* [85–87]. Although their direct role in *NPR1* ubiquitination has not been reported, their transcript levels, especially those of *ATL2* and *PI4KG4*, have been shown to increase following exposure of plants to chitinases [88] and treatment with SA [89], respectively. The role of these proteins during drought stress has equally been demonstrated [85]; and proteins, such as *XBAT34* have been shown to be induced by *6-BA* (*6-benzylaminopurine*) and SA [90]. Additionally, *XBAT34* contains two Ankyrin repeats, which are also core motifs found in *NPR1* protein and are important in mediating interactions with other proteins [58, 90–92]. The importance of the *ankyrin* repeats within the *NPR1* sequence is elegantly demonstrated in the inability of *npr1* mutants to activate PR genes following pathogen attack due to a distortion in this region [58, 93]. A distorted *ankyrin* repeat within the *NPR1* protein hampers its ability to bind to transcription factors belonging to the basic leucine zipper (*BZIP*) family such as *TGA3* (Cluster 1A) for a positive defence outcome [92, 94–97].

Two other *BZIPs* (*BZIP60* and *BZIP28*) were identified in this study and have been implicated in *Unfolded Protein Response* (UPR) during stress response [98–100]. The ability of the *BZIP* domain to translocate to the nucleus during stress could be a key contribution to its role during stress responses. In fact, although we did not find a direct relation between *BZIP28* and *BZIP60* to *NPR1* in literature, existing evidence shows that the *inositol-requiring protein-1a* (*IRE1a*)/*BZIP60* branch of the UPR pathway have a role in PR protein secretion following treatment with SA [99]. In addition to the *BZIP* transcription factor (*TGA3*) identified here, proteins belonging to the *WRKY* family of transcriptional factors [101] – *WRKY70* (Fig. 3; Cluster 1A) and *WRKY40* (Fig. 3; Cluster 2) were equally identified. Unlike *TGA3*, no direct interaction between *NPR1* and *WRKY40* was found during our literature search. However, similar to *NPR1* monomers, evidence for the nuclei localization of *WRKY40* does exist [102]. Its role in stress response has also been demonstrated and, its ability to interact with other *WRKY* proteins has been shown [102, 103]. For instance, co-expression of *WRKY18* and *WRKY40* led to increased susceptible of plants to *P. syringae* and *Botrytis cinerea* correlating with reduced PR gene expression [102]. *WRKY70* on the other hand is known to positively mediate pathogen defence response in an SA-dependent fashion which is independent of the *NPR1* pathway [104]. *PAD4*, which is another protein identified (Cluster 1A), can act dependently (e.g., for SA-dependent defence response) or independently (e.g., for basal immunity [105]) of *NPR1* for PR-1 gene expression [106, 107]. *NPR1* also has the ability to interact with non-transcription factors, such as *NIMIN-1*, identified in this study (Cluster 1A) and experimentally verified to bind to *NPR1* [108, 109] to modulate PR-1 gene expressions during SAR.

#### Retrieving enriched processes and pathways mediating the *Arabidopsis thaliana* plant defence

A total of 114 biological processes were found to be involved in the *Arabidopsis thaliana* protection and defence mechanisms, among which 74 were found to be non-redundant considering the feature of the GO structure [47]. 21 processes were identified to be statistically significant in plant defence mechanisms and Table 2 provides a summary of the type of response and biological activities in which these enriched processes are involved. These processes are related to both biotic and abiotic triggered responses, leading to the initiation of major pathways involved in the innate response (GO:0010200), HR (GO:0009626) and SAR (GO:0009862) in plants [2–7]. In general, these processes are triggered on the basis of the (pathogen) attacks classified using the mode of plant-pathogen interactions. For example, a pathogen may survive by using the living plant cells (for biotrophic pathogens) or by killing plant cells and feeding on these dead cells (for necrotrophic pathogens) [110]. Interestingly, the enrichment pathway analysis reveals two enriched KEGG pathways participating to *NPR1*-associated plant defence response, namely *Plant-pathogen interaction* (http://www.genome.jp/kegg-bin/show_pathway?ath04626) and *Plant hormone signal transduction* (http://www.genome.jp/kegg-bin/show_pathway?ath04075) with *p*-values of 0.013085 and 0.02070, respectively. These pathways show evidence of regulating a wide variety of enriched biological processes, including *programmed cell death* (PCD) and defence, HR, ABA and SA dependent responses.

**Table 2:** Statistically significant or enriched biological processes protecting the *Arabidopsis thaliana* plant

GO ID	Process name	L	*p*-value	ap-value	Process functions	Stressor	Tps	RF
GO:0010200	*Response to chitin*	5	1.40*e*−11	1.04*e*−09	Defence against chitin bearing pathogens such as fungi, exoskeletons of animals and nematodes.	Biotic	Biotrophics	*✓*
GO:0000165	*MAPK cascade*	9	7.69*e*−12	5.69*e*−10	The process involves programmed cell death as a way of responding to biotic and abiotic factors.	Biotic	Biotrophics	*✓*
GO:0006612	*Protein targeting to membrane*	8	0.0	0.0	Directs the proteins to the cell membrane for further transportation and communication of chemicals.	Biotic & abiotic	-	✗
GO:0001666	*Response to hypoxia*	5	8.12*e*−09	6.01*e*−07	The response is triggered when the oxygen goes below, 20.8−20.95*%*.	Abiotic	-	✗
GO:0009611	*Response to wounding*	3	8.61*e*−06	6.37*e*−04	A biological mechanism that takes place indicating the damage of an organism.	Biotic & abiotic	Biotrophics	*✓*
GO:0009738	*Abscisic acid (ABA)-activated signalling pathway*	7	0.0	0.0	Controls environmental stress for example, drought and plant pathogens [115].	Abiotic & biotic	Necrotrophics	*✓*
GO:0009409	*Response to cold*	4	0.0	0.0	Reacts to changes in low temperatures.	Abiotic	-	✗
GO:0061025	*Membrane fusion*	4	0.0	0.0	Ensures life continuity in organisms.	Biotic & abiotic	Necrotrophic & biotrophics	*✓*
GO:0009626	*Plant-type hyper-sensitive response*	6	5.29*e*−12	3.91*e*−10	Killing of cells surrounding the infected area (programmed cell death) to starve pathogens like biotrophics	Biotic	Biotrophics	*✓*
GO:0010310	*Regulation of hydrogen peroxide metabolic process*	6	3.51*e*−11	2.60*e*−09	Controls plant stress and programmed cell death. It is also a go-between the wounding responses and biotic interactions.	Biotic & abiotic	Necrotrophics	*✓*
GO:0009697	*Salicylic Acid biosynthetic process*	9	2.72*e*−11	2.01*e*−09	Forms salicylic acid which is a fungicide present in some plants.	Biotic	Biotrophics	*✓*
GO:0002679	*Respiratory burst involved in defense response*	4	2.86*e*−11	2.12*e*−09	Ensures life continuity in plants when the defence response consumption rate increases for instance, the presence of phagocytic leukocytes.	Biotic	Necrotrophics & biotrophics	*✓*
GO:0035304	*Regulation of protein dephosphorylation*	8	0.0	0.0	Controls the removal rate of phosphorous, suppresses pathogenesis related expression and promotes programmed cell death.	Biotic	Biotrophics	*✓*
GO:0031348	*Negative regulation of defence response*	6	0.0	0.0	Controls the frequency of the defence response.	Biotic	Necrotrophics	*✓*
GO:0010363	*Regulation of plant-type hyper-sensitive response*	7	1.01*e*−11	7.51*e*−10	Balances the hyper-sensitive response.	Biotic	Biotrophs	*✓*
GO:0009862	*Systemic acquired resistance, salicylic acid mediated signalling pathway*	8	4.99*e*−12	3.70*e*−10	Salicylic acid plays a vital role in the expression of pathogenesis related proteins.	Biotic	Biotrophics	*✓*
GO:0043069	*Negative regulation of programmed cell death*	6	0.0	0.0	Stops the occurrence of programmed cell death.	Biotic	Necrotrophics	*✓*
GO:0050832	*Defence response to fungus*	6	2.41*e*−11	1.78*e*−09	Protects the cell plants from fungi.	Biotic	Necrotrophics & biotrophics	*✓*
GO:0009410	*Response to xenobiotic stimulus*	3	4.06*e*−06	3.00*e*−04	Takes place when the system identifies foreign compounds in the organism.	Biotic	Necrotrophics & biotrophics	*✓*
GO:0010112	*Regulation of systemic acquired resistance*	7	1.70*e*−04	0.0126	Balancing of systemic acquired resistance.	Biotic	Biotrophics	*✓*
GO:0016045	*Detection of bacterium*	6	4.11*e*−11	3.04*e*−09	Converts the signal of presence of bacteria into a molecular signal.	Biotic	Necrotrophics & biotrophic	*✓*

For each process identified, following information is provided: Level (L) in the GO directed acyclic graph structure, *p*-value and adjusted *p*-value (ap-value), functional activities triggered, nature of stress thwarted, type of pathogens suppressed (Tps) and whether it contributes to response to fungi (RF) or not

**Fig. 4:**
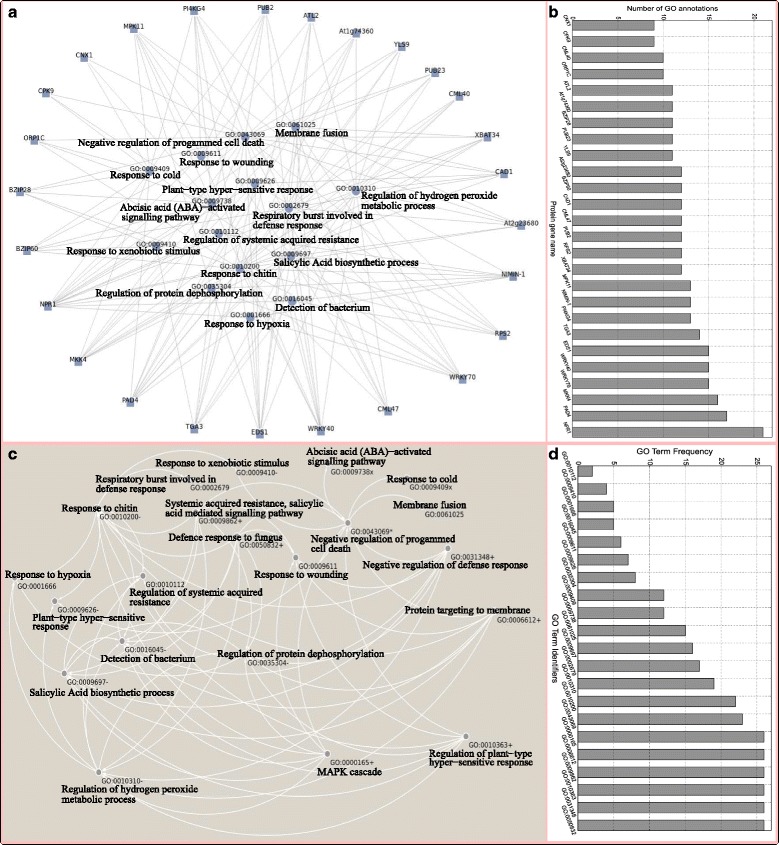
Summary results of the biological mechanism of the *NPR1*-based defence response. **a** plotted using the PINV tool [113] is a protein-process map showing *NPR1*-associated plant defence proteins and enriched processes in which they are involved, and the number of enriched processes in which each protein is involved is shown in (**b**). Note that processes in which all proteins are involved are not displayed in (**a**) to avoid that high number of links limits visibility. **c** plotted using the Cytoscape tool [114] displays *NPR1*-based process regulatory network, showing possible process occurrence sequences and (**d**) shows the number of proteins involved in or frequency of each enriched process. The suffix +, ∗, ×, or − were added to enriched process according to the fact all *NPR1*-associated plant defence proteins are involved in the process (+), or set of proteins involved in the process contains all *NPR1*-associated plant defence proteins are implicated in the *Plant-pathogen interaction* (×) or *Plant hormone signal transduction* (−) or both (∗) pathways

*NPR1*-associated plant defence proteins share six processes, namely *MAPK cascade* (GO:0000165), *SAR, SA mediated signalling pathway* (GO:0009862), *Regulation of plant-type hyper-sensitive response* (GO:0010363), *Protein targeting to membrane*(GO:0006612), *Defence response to fungus* (GO:0050832) and *Negative regulation of defence response* (GO:0031348). The number of processes in which each protein is contributing is shown in Fig. 4 b and a, highlighting different protein-process associations. Interestingly, *NPR1* is involved in all 21 enriched processes identified, suggesting that *NPR1* is a potential regulator of the plant ubiquitin proteolytic system during infection, controlling associated protein expression levels and functioning at the cross-roads of several signalling pathways, as well as modulating antagonistic cross-talk between different signalling pathways [83, 97] critical to the plant defence-mediated response. This suggests that the success of the plant defence mechanism is a result of these different concerted and controlled signal transduction events that sequentially occur in the plant system, leading to an increased resistance to diverse attacks. As a consequence, the failure of these processes to function properly may yield increased susceptibility to the pathogen, which possibly negotiates its entry into the cell by activating processes to thwart plant defence mechanisms. Thus, we built a potential enriched process-based regulatory network using the following binary relation rule: *Given two processes s and t, the process t is triggered after the process s, which is indicated in the regulatory network by an arrow from s to t, if* *A*_*t*_ *⊂* *A*_*s*_ *and* $A_{s} \supseteq \mathcal {P}.$ Under the assumption that a process is triggered after another as a result of specific degradation of a single or a subset of proteins, and where *A*_*x*_ is the set of proteins participating to the process *x* and $\mathcal {P}$ is the set of proteins in at least one of the enriched pathways identified.

Figures 4 c and d show a potential enriched process-based regulatory network constructed and process frequencies (number of proteins annotated by each process), respectively. This regulatory network reveals, for example, that there are only five possible processes that may be triggered following the occurrence of the *negative regulation of PCD* (GO:0043069), namely: *Response to xenobiotic stimulus* (GO:0009410), *Membrane fusion* (GO:0061025), *Plant-type hyper-sensitive response (HR)* (GO:0009626), *Abscisic acid (ABA)-activated signalling pathway* (GO:0009738) and *Response to cold* (GO:0009409) processes. Most of these processes are expected to occur after the occurrence of the *negative regulation of PCD*, which could be triggered after the occurrence of processes, such as *Negative regulation of defence response* (GO:0031348). This is consistent as the system may trigger mechanisms susceptible to promote or normalize the plant defence response by coordinating different actions in order to offer appropriate response to and to contain the infection. For example, HR and ABA-activated signalling pathway may relaunch new PCD by killing the infected cells at the point of invasion [2, 6, 7, 111]. This regulatory network also reveals that the occurrence of the *negative regulation of PCD* can result from the dysfunction of critical processes, such as *MAPK cascade* (GO:0000165), *Protein targeting to membrane* (GO:0006612) and *regulation to hydrogen peroxide* (GO:0010310), which are vital for the regulation of a wide variety cellular activities, including transportation and communication of chemical materials, proliferation, differentiation, apoptosis and stress response [112].

## Conclusions

This study proposes a novel bioinformatics protocol that can be readily applied to plant phenotype based gene knock-out or -down experimental dataset to assess the contribution of the gene under consideration to plant-defence mechanisms and to build a potential enriched biological process-based regulatory network using publicly available biological data. This protocol enables the extraction of the specific (differentially infected) samples of the experimental dataset to eliminate potential artefacts, such as uncertainty of the data set and potential noise inherent in the experiment, which could mask downstream analysis. We applied the protocol to the phenotype dataset expressed in terms of leaf spore counts of 60 *npr1* gene mutant and wild-type *Arabidopsis thaliana* plants following *Pst*-DC3000 infection. Results obtained still showed that *NPR1* plays an important role during plant pathogen response by suppressing the growth of the pathogen. More specifically, we found that differences in the plant susceptibility to the *Pst*-DC3000 infection depend on whether the *NPR1* protein is functionally active or not with about 99% of variability in *Pseudomonas* spore growth between *npr1* mutant and Wt plant samples. The increased disease susceptibility phenotypes observed in *npr1* mutant plants following to *Pst*-DC3000 infection may be due to the fact that the SAR signalling pathway, which is essential for plant defence response, is blocked in these plants as a result of knocking out or down the protein *NPR1*, leading to the attenuation of PR gene expressions. This SAR signal requires a functionally active *NPR1*, which is a key regulatory protein of SAR, acting as a controller and modulator of PR gene expressions.

With the increasing number of publicly available biological data which already demonstrates the complexity of the plant defence network, new computational approaches are essential to provide an easy-to-analyse picture which can lead to the identification of gaps and opportunities for the development of future ‘wet or dry lab’ experiments. The proposed protocol uses the GO-universal metric based Gene Ontology semantic similarity model to identify putative proteins collaborating with *NPR1* in regulating plant defence mediated response and retrieve enriched biological processes involved. We identified 26 *NPR1*-associated plant defence proteins and 21 highly specific processes, which related to the major forms of defence processes reported (innate response, HR, SAR, etc.), and construct the potential process-based regulatory network, predicting occurrence sequences of different processes. This *NPR1*-based enriched process regulatory network (Fig. 4 c) can effectively reveal eventual sequences of biological processes occurring during the plant defence response. As illustration, it suggests that the suppression of the SAR signalling pathway (GO:0009862) may thwart processes that enable the detection of the pathogen (GO:0016045), control plant stress, promote PCD and attenuate PR gene expressions (e.g., GO:0010310, GO:0035304) to favor processes leading to the *negative regulation of PCD* (GO:0043069) in order to trigger processes that possibly ensure pathogen life continuity within plant cells. Different results obtained demonstrate that this novel protocol is effective for assessing plant-defence genes and may help better understand the plant defence mechanisms, an important aspect in the biology of plants.

## Additional files

Additional file 1 Experimental data from *Arabidopsis* wild type and *npr1* mutant plants. Leaf bacteria spore count (phenotype) dataset of 31 *Arabidopsis* wild type (control) and 29 *npr1* mutant (case) plants 48 h post *Pst*-DC3000 infection. (XLS 10 kb)

Additional file 2 Checking normality assumption using Q-Q plots. Checking normality assumption for initial and transformed differentially infected wild and *npr1* mutant plant spore count datasets using Q-Q plots. (PDF 536 kb)

## Abbreviations

ABA: Abscisic acid
ANOVA: Analysis of variance
DCD: Development and cell death
dof: Degree of freedom
ES: Aggregated score
Et: Ethylene
GO: Gene ontology
GOA: Gene ontology annotation
HR: Hypersensitive response
ICS: Individual closeness score
ISR: Induced systemic response
JA: Jasmonic acid
KEGG: Kyoto Encyclopeadia of genes and genomes
*NPR1*: *non-expressor of pathogenesis-related 1*
P. syringae: Pseudomonas syringae
PAMP/MAMP: Pathogen associated molecular patterns/microbe-associated molecular pattern
PCD: Programmed cell death
post-GSB: Post-gene silencing bioinformatics
PPI: Protein-protein interaction
PR: Pathogenesis-related
Pst-DC3000: P. syringae pathovar tomato strain DC3000
Q-Q: Quantile-Quantile
R: Resistance
SA: Salicylic acid
SAR: Systemic acquired resistance
UPR: Unfolded protein response
Wt: Wild type
