# Supplementary material for: A post-gene silencing bioinformatics protocol for plant-defence gene validation and underlying process identification: case study of the Arabidopsis thaliana NPR1
Source: BMC Plant Biol. 2017 Nov 23;17:218. doi: 10.1186/s12870-017-1151-y (PMC5701366; doi:10.1186/s12870-017-1151-y)
Supplement: Supplementary file 2 — Checking normality assumption using Q-Q plots. Checking normality assumption for initial and transformed differentially infected wild and npr1 mutant plant spore count datasets using Q-Q plots. (PDF 536 kb) [file 12870_2017_1151_MOESM2_ESM.pdf]

Checking normality assumption for initial and transformed differentially infected wild and npr1 mutant plant spore count datasets using Q-Q plots

Rosita E. Yocgo[1, 2], Ephifania Geza[1, 3], Emile R. Chimusa[4], and Gaston K. Mazandu[1, 2, 3*]

[1]*African Institute for Mathematical Sciences (AIMS), Cape Town, South Africa & Cape Coast, Ghana.*

[2]*Biomathematics Division, Department of Mathematical Sciences, Stellenbosch University, South Africa.*

[3]*Computational Biology Group, Department of Integrative Biomedical Sciences, Institute of Infectious Disease and Molecular Medicine, University of Cape Town, Medical School, Anzio Road, 7925 Observatory, South Africa.*

[4]*Division of Human Genetics, Department of Pathology, Institute of Infectious Disease and Molecular Medicine, University of Cape Town, Medical School, Anzio Road, Observatory 7925, South Africa.*

---

*To whom correspondence should be addressed.[1]

[1]Email: kuzamunu@aims.ac.za

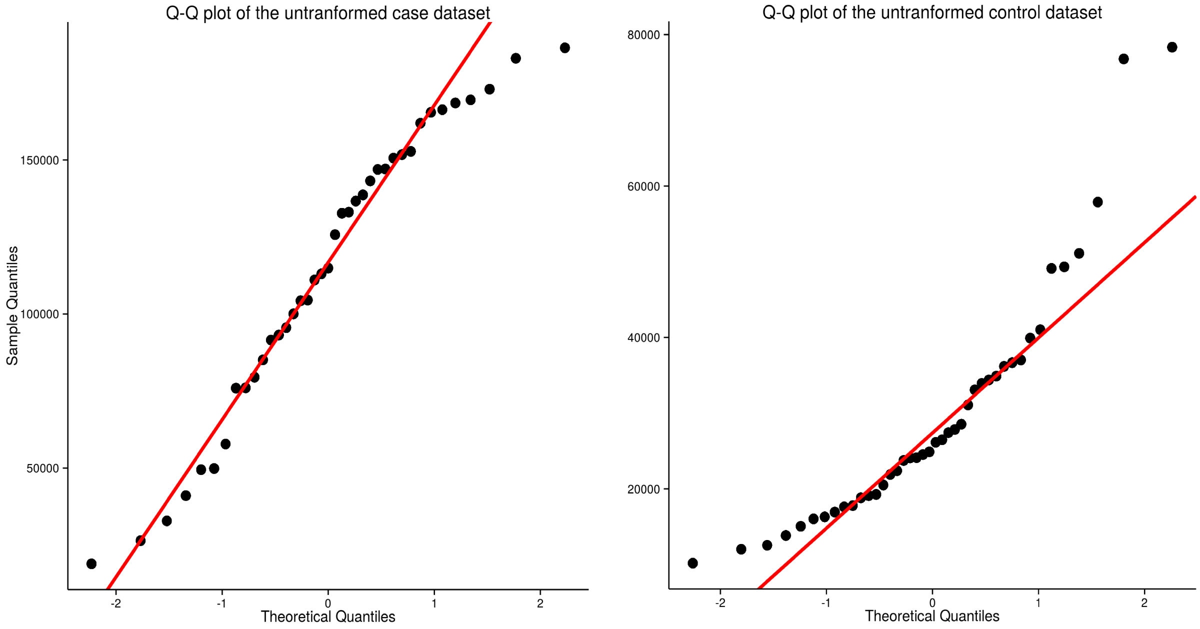

Figure S1: **Q-Q plots of differentially infected plant dataset Before transformation.** For the case dataset (Figure on the left side), data points are closely clustered along the fitted line, indicating that the case dataset are normally distributed, whereas in the control dataset (Figure on the right side) data points are relatively far from the fitted line revealing that the control dataset is not normally distributed as confirmed by the Shapiro-Wilk test.

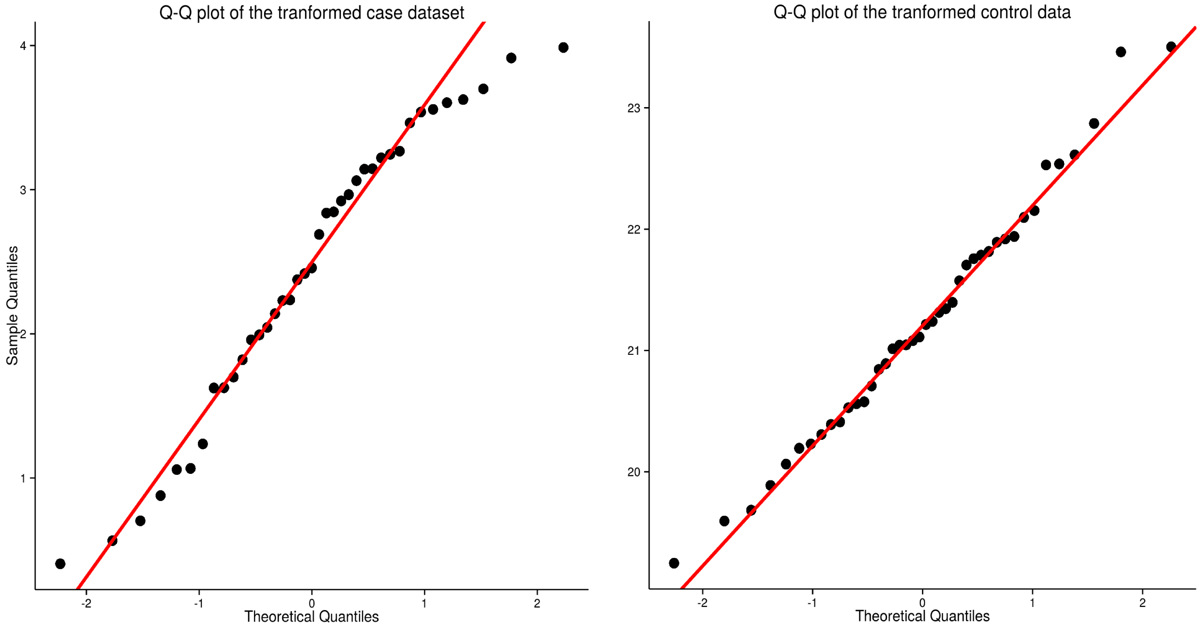

Figure S2: **Q-Q plots of differentially infected plant dataset after transformation.** For the two datasets (case and control on the left the right sides, respectively, data points are closely clustered along the fitted line, indicating that these two datasets are now normally distributed, thus satisfying requirement for applying the analysis of variance (ANOVA) method or one-way test depending on whether the two datasets have equal variance or not.
